# Prurigo Nodularis Eruption Triggered by SARS-CoV-2 Vaccine

**DOI:** 10.7759/cureus.29914

**Published:** 2022-10-04

**Authors:** Ghassan Barnawi, Fareeda S Alghamdi, Amer S Almuqati, Awadh Alamri

**Affiliations:** 1 Dermatology, King Abdulaziz Medical City, Jeddah, SAU; 2 Plastic and Reconstructive Surgery, King Abdulaziz Medical City, Jeddah, SAU

**Keywords:** biopsy, immunization, covid-19, pruritis, prurigo nodularis (pn)

## Abstract

We discuss a case of a 63-year-old male who presented with generalized itchy papulonodular rash a few weeks after receiving a vaccination against SARS-CoV-2. The patient had a negative medical history for atopic dermatitis and other pruritic skin conditions, and clinical presentation was consistent with prurigo nodularis, which was confirmed later by tissue biopsy and microscopic analysis. The pathophysiology of this skin condition is thought to be due to an overlap between the immune and nervous systems. Due to the hypothesized involvement of the immune system in this disease, it. is presumed that the patient had a dysregulated immune response caused by his recent SARS-CoV-2 vaccination.

## Introduction

Pruritus is an unpleasant sensation that causes the desire to scratch and can be a debilitating symptom associated with many primary and secondary skin disorders [1]. Chronic pruritis can lead to the development of other pruritic secondary skin lesions, which will lead to a continuous itch-scratch cycle [2]. The two most common secondary skin lesions associated with chronic pruritis are lichen simplex chronicus and prurigo nodularis (PN) [2]. PN presents as papular and nodular lesions with excoriations, ulceration, and post-inflammatory hyper-pigmentary changes [1]. An interplay between the nervous and immune systems has been thought to be involved in the pathogenesis of PN [3]. Certain dermatological and non-dermatological conditions have been frequently associated with PN such as diabetes mellitus type 2 and atopic dermatitis [3]. In addition, emerging scientific evidence has reported PN as one of the rare skin-related complications seen in patients receiving the SARS-CoV-2 vaccination [4]. This report discusses the case of a 63-year-old gentleman who presented to the dermatology department with skin lesions consistent with PN after he received his first dose of the Pfizer-BioNTech SARS-CoV-2 vaccine. 

## Case presentation

A 63-year-old gentleman was referred to the dermatology clinic in October 2021 due to generalized itchy skin lesions. The rash started five months prior to the day of the office visit, and it appeared initially as erythematous, painful, and itchy papules over the bilateral lower limbs and gradually progressed to include the whole body sparing the neck and face. Lesions were associated with intermittent attacks of severe pruritus that lasted for approximately 30 minutes. The patient reported that the onset of symptoms started two weeks following the first dose of the Pfizer-BioNTech vaccine, which he received in April 2021. However, he denied any new skin lesions or worsening of previous lesions with subsequent booster doses of the same vaccine. Past medical history was negative for similar skin manifestations in relation to food, medications, or contact with animals. Past medical history was positive for type 2 diabetes mellitus for 30 years, which has been managed by only oral anti-glycemic medications with a recent hemoglobin A1C of 9. The patient did have a history of atopy or other pruritic skin conditions, and screening for thyroid stimulating hormone (TSH), liver function tests (LFTs), and estimated glomerular filtration rate (eGFR) was normal. Reviewing the patient's primary care doctor's notes indicated that he had no history of any psychiatric conditions or cognitive decline. Several over-the-counter moisturizing creams, topical herbal treatments, and honey were used by the patient to relieve the itchiness and rash without any positive outcomes. The rash was severe enough to cause disturbance in his sleep pattern and quality; however, his daily activities were not affected.

**Figure 1:**
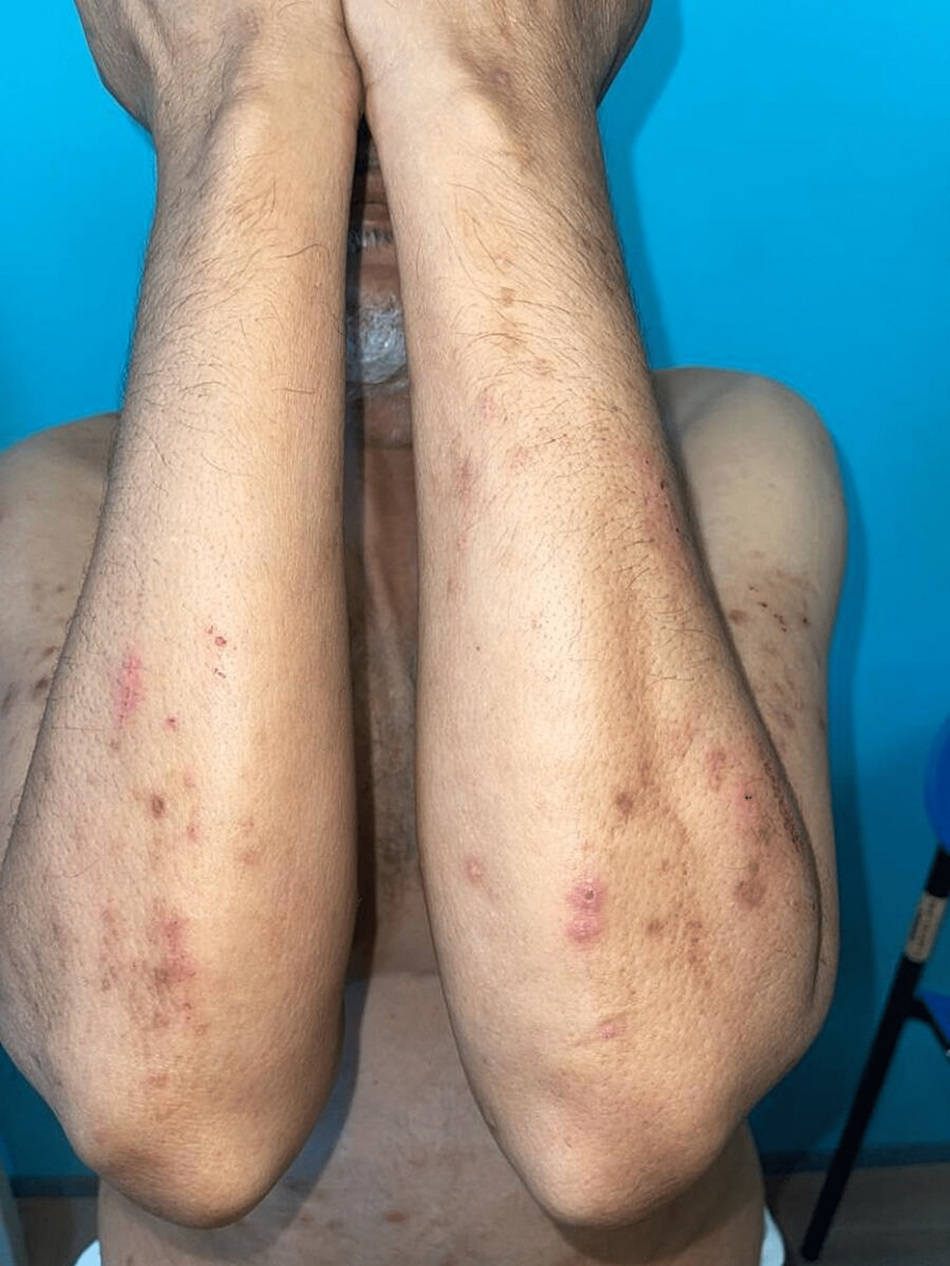
Extensor forearms showing erythematous, excoriated papules, and post-inflammatory hyperpigmentation.

**Figure 2:**
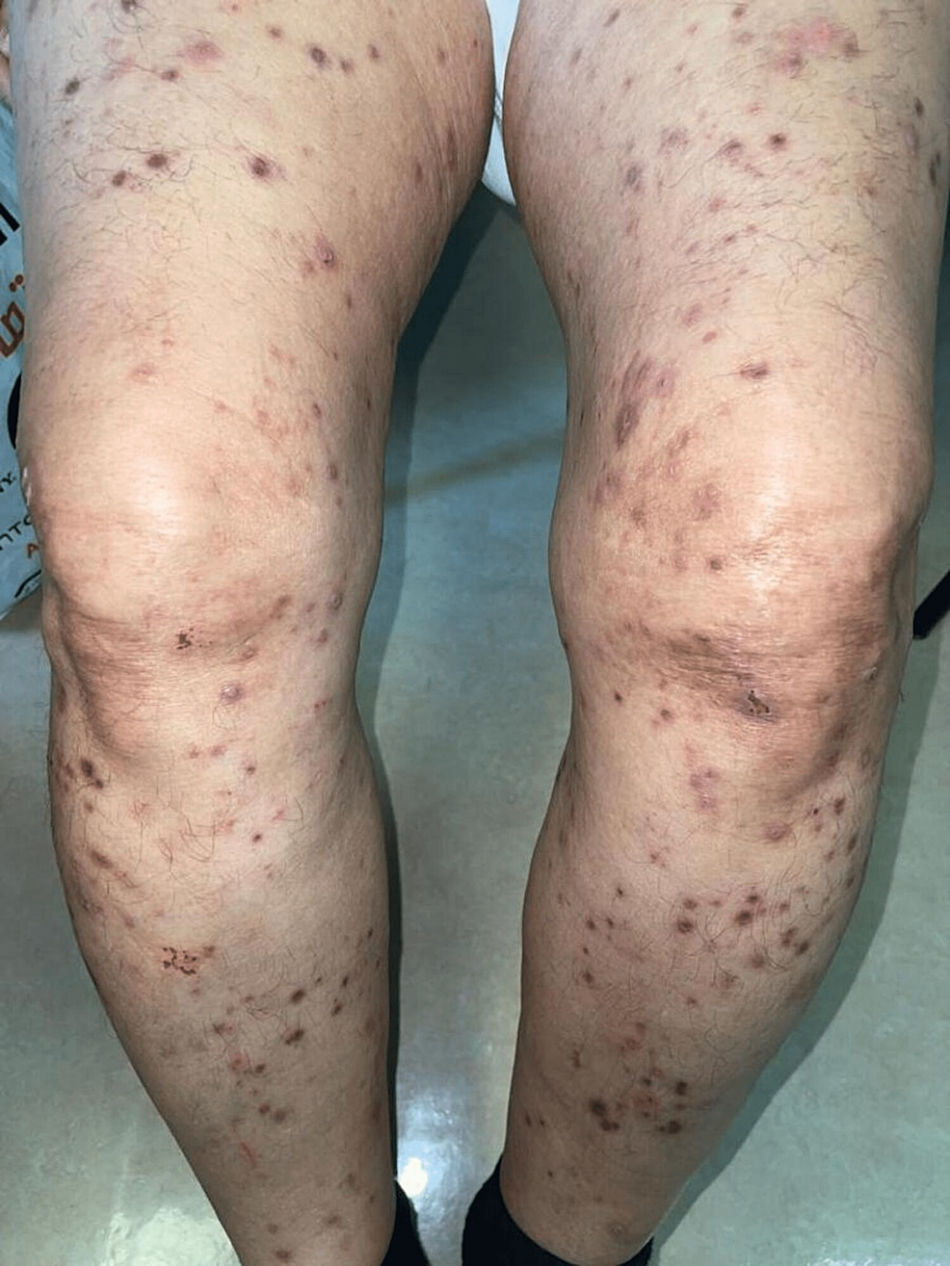
Widespread erythematous and brown lichenified papular lesions in the extensor side of both lower limbs.

**Figure 3:**
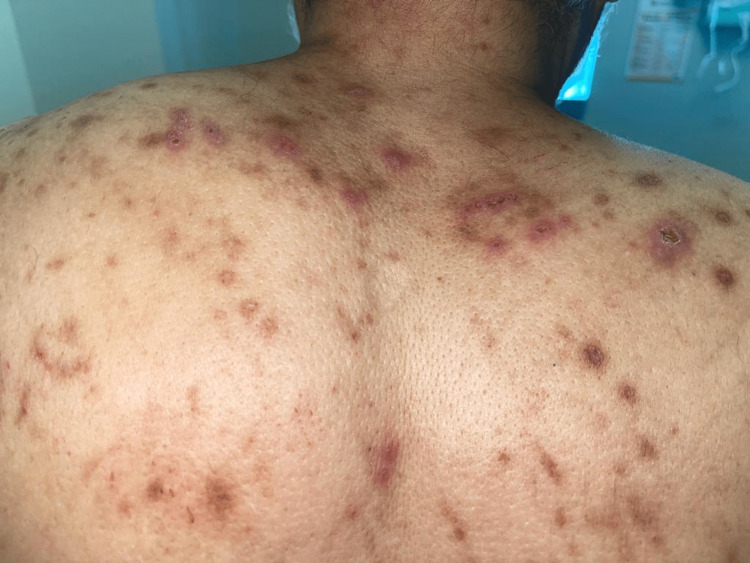
Upper back showing excoriated papules and nodules in a butterfly distribution.

**Figure 4:**
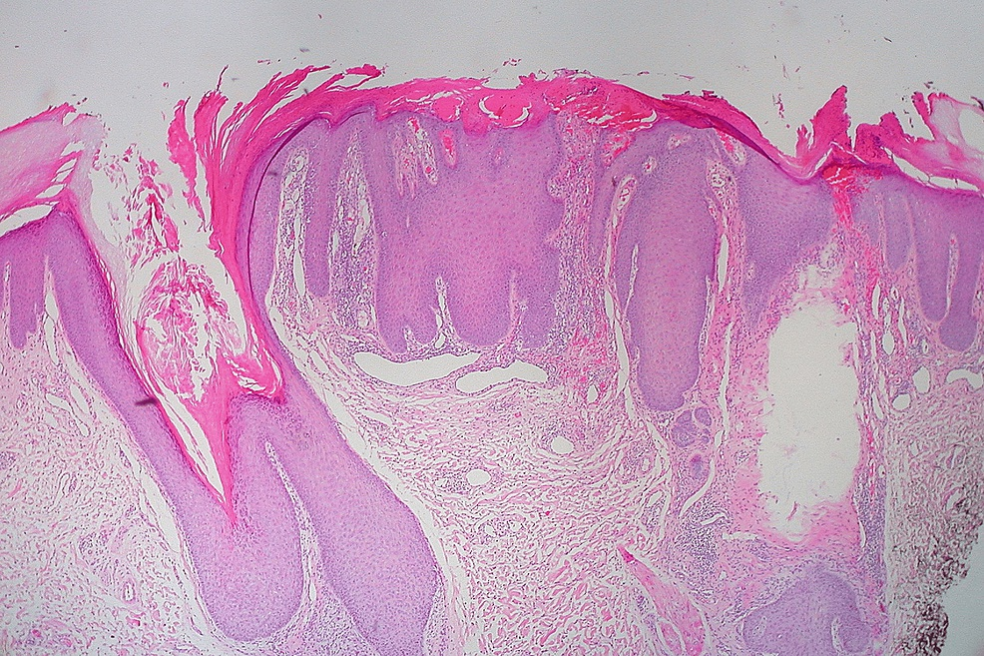
Histopathology of prurigo nodularis with H&E stain. H&E: hematoxylin and eosin

On examination, the patient had erythematous and brown papulonodular lesions associated with varying degrees of hemorrhagic crusting, erosions, and ulceration. Lesions were widespread and involved extensor upper (Figure 1) and lower (Figure 2) extremities and were symmetrically distributed over the upper back with a classic butterfly distribution (Figure 3) ). A 4 mm punch biopsy was performed on two active lesions in different locations, and two tissue samples were sent for histopathological analysis. Histopathology showed irregular acanthosis, hypergranulosis, and elongated rete ridges consistent with chronic picking and scratching of the skin (Figure 4). The patient's clinical lesions and histopatholgical findings were consistent with the diagnosis of PN. He was started on topical steroids and emollients including QV cream (Ego Pharmaceuticals, Braeside, Victoria, Australia) and petroleum jelly, and was prescribed oral antihistamines for initial treatment. Due to the extent of involvement, he was scheduled to receive narrowband ultraviolet B (NUVB) phototherapy as well. 

## Discussion

Pruritus is defined as an unpleasant sensation that causes the desire to scratch, and it is the most common skin-related symptom [1]. The itch sensation can be localized to the site pathology or generalized, and symptoms might be intermittent or constantly eliciting the desire to scratch [1]. Pruritus is mostly due to a primary skin disorder, but up to 25% of cases can be caused by systemic diseases [5]. The most common primary skin disorders that cause pruritis include xerosis, eczematous dermatitis, urticaria, and papulosquamous disorders [2]. On the other hand, systemic causes of pruritus present without visible skin lesions and are mostly due to malignancy and hepatic or renal dysfunction, which leads to the accumulation of waste and metabolic byproducts known to cause itchy skin [2].

Chronic rubbing and itching from either primary skin lesions or systemic diseases can lead to the development of other skin disorders such as lichen simplex chronicus and PN, which tend themselves to be pruritic and lead to a continuous itch-scratch cycle [2]. PN is a reactive inflammatory skin condition that presents with papular or nodular lesions associated with excoriation or ulceration [3]. Lesions are typically found in a symmetric bilateral distribution of the body extensors with sparing of the face and groin [3]. The exact pathogenesis of the disease remains unclear, but immunologic and neuronal dysregulation is thought to be an integral mediator of the pathophysiology of PN [3]. Patients who present with PN usually have a positive history of atopic dermatosis, but other secondary causes of pruritus including psychiatric conditions should be investigated as well [3]. Common laboratory tests ordered for patients with PN include TSH, liver enzymes, and renal function tests [2]. In addition, the SARS-CoV-2 vaccine has been reported to be a possible trigger for the development of multiple dermatologic conditions including PN [4]. Due to the important role of the immune system in the pathogenesis of PN, it was thought that our patient had an immune dysregulation elicited by his SARS-CoV-2 vaccination [4].

Diagnosis is made clinically based on the patient's history and physical exam with the presence of characteristic PN lesions [2]. A biopsy is not routinely indicated for diagnosis and should be considered if a patient fails initial treatment or if the diagnosis could not be confirmed clinically [2]. Histopathology of these lesions shows epidermal hyperplasia with hypergranulosis, overlying compact hyperkeratosis and sparse papillary dermal perivascular lymphocytic infiltrate [6]. Treatment of PN can be challenging and multifactorial to address the different contributory factors to this disease [2]. Managing an identified underlying cause for pruritus in these patients should be the first step [2]. Generally, all patients must receive education about the importance of proper skin hygiene including the use of recommended bathing cleansers and body moisturizers to avoid skin irritation and xerosis, which represent the most common causes of purities [2]. A first-generation oral antihistamine can be considered to further reduce symptoms of pruritus [2]. For patients with limited or localized disease, treatment can be achieved with topical or intralesional steroids [3]. Phototherapy with NUVB is the mainstay of treatment for patients with widespread involvement [2]. Other systemic treatments can be utilized for those who fail or have contraindications to light therapy [2]. They include systemic immunosuppressants, thalidomide, lenalidomide, and anticonvulsants [2].

## Conclusions

PN is a disorder with a multifactorial etiology. The immune system plays a crucial role in the pathogenesis of PN, and dysregulation in the immune system elicited by the SARS-CoV-2 vaccine can trigger the development of this condition in predisposed individuals. It is important to continue to further investigate the pathophysiology of this skin condition and its association with the SARS-CoV-2 vaccine. 
